# Understanding the Use of Composite Endpoints in Clinical Trials

**DOI:** 10.5811/westjem.2018.4.38383

**Published:** 2018-06-04

**Authors:** C. Eric McCoy

**Affiliations:** UC Irvine School of Medicine, Department of Emergency Medicine, Orange, Californua

## Abstract

Clinicians, institutions, healthcare networks, and policymakers use outcomes reported in clinical trials as the basis for medical decision-making when managing individual patients or populations. Therefore, the choice of a valid primary endpoint is crucial for randomized controlled trials (RCT) to demonstrate efficacy of new therapies. Recent improvements in treatment, however, have led to a decline in the morbidity and mortality of several common diseases, resulting in a reduction in relevant outcomes that can be used as clinical trial endpoints. Composite endpoints have been used as a solution to maintain the feasibility of RCTs, particularly when facing low event rates, high cost, and long follow-up. However, the benefits of using composite endpoints must be weighed against the risks of misinterpretation by clinicians and policymakers, as incorrect interpretation may have a detrimental effect on patients and populations. This paper defines a composite endpoint, discusses the rationale for its use, and provides a practical approach to interpreting results to aid in medical decision-making.

## INTRODUCTION

Advances in medicine have led to decreased morbidity and mortality for many common medical conditions, with overall improvement in health of the population.^1^–^2^ The smaller marginal benefit of new treatments has provided increasing challenges for medical research as smaller incremental benefits (effect sizes) of new therapies typically require studies with larger sample sizes and longer follow-up, both of which can be cost prohibitive.^3^–^5^

Researchers have been increasingly using composite endpoints in lieu of the customary single primary endpoint.^6^,^7^ Although statistically treated like a single primary endpoint, composite endpoints provide unique challenges for patient care.^6^–^8^ If used or interpreted incorrectly, they have the potential for detrimental impact on patient care on a large scale. This paper defines composite endpoints, discusses the rationale for their use, and provides a practical approach to understand whether they should be used in medical decision-making.

### Composite Endpoint Defined

A composite endpoint consists of at least two or more distinct endpoints, called component endpoints.^6^,^7^ Because of the need to observe a certain number of primary endpoints to achieve adequate statistical power for a study, investigators opt to use component endpoints that contribute to an overall composite event rate.^9^

### Rationale for Using Composite Endpoints in Clinical Trials

#### Benefits

Combining two or more study outcomes into a single composite measure typically results in an increase in the incidence rates of the composite endpoint and improves the ability to detect differences in the primary endpoint. This pooling of different study outcomes will result in higher event rates and increased statistical precision that will subsequently lead to designing clinical trials that include fewer patients, are less costly, and can be completed in a more timely manner.^8^,^9^ Other reported benefits include avoiding competing risks in outcome assessment and addressing rare instances where there is no obvious choice of a primary trial outcome.^10^,^11^

#### Challenges

Interpretation of composite endpoints remains difficult, as there are no generally accepted standardized approaches to interpretation, and evaluating a composite endpoint as if it were a single primary endpoint is an inadequate strategy. To date, there remains little guidance available on how these aggregated endpoints should be interpreted.^6^

One common challenge that arises in the process of interpretation is how to evaluate a composite outcome that is composed of component endpoints that may not be clinically meaningful. Combining component endpoints with large variability in importance (to patients or clinicians) raises substantial concerns when attempting to use a composite endpoint as a basis for medical decision-making. Similar concerns arise when there is a large gradient in the frequency of the most and least important component outcomes, as well as in the variability of point estimates of the component outcomes.

### Interpreting Composite Endpoints

The ultimate question that clinicians must answer when evaluating studies that use composite endpoints is whether or not the composite endpoint should be used as a basis for medical decision-making. The following section contains critical foundational questions that must be answered when considering the use composite endpoints.^7^ To the extent that one can answer “yes” to the following questions, one can feel confident using the treatment effect on the composite endpoint as the basis for medical decision-making. Conversely, to the extent that one answers “no” to the following questions, one should use the individual component endpoints instead (Figure).

### Are the Component Endpoints of Similar Importance to Patients?

If the component endpoints of a composite endpoint are of equal (or relatively similar) importance to patients, then it does not matter how a relative risk reduction is distributed among the components because if the composite crossed the threshold for statistical significance, one can be assured that an important component played a substantive role. The larger the gradient in importance between the most and least important component endpoints, the larger our skepticism about the usefulness of the composite endpoint.

An illustrative example of how an increase in the importance gradient leads to increased skepticism regarding using a composite endpoint can be seen in a study that evaluated the effect of systemic glucocorticoids on chronic obstructive pulmonary disease (COPD).^12^ In this prospective, double-blind, randomized controlled trial, the authors evaluated the effectiveness of systemic glucocorticoids vs. placebo on the primary endpoint of treatment failure, which was a composite endpoint of death from any cause, need for intubation, readmission to the hospital for COPD, or intensification of drug therapy, for patients presenting with COPD exacerbations. The authors report that the rates of treatment failure were significantly higher in the placebo group at 30 days (33% vs. 23%, p=0.04) and 90 days (48% vs. 37%, p=0.04). The authors concluded that treatment with systemic glucocorticoids resulted in moderate improvement in clinical outcomes among patients hospitalized for exacerbations of COPD.

Combining an endpoint of paramount clinical importance such as death with a component endpoint of relatively trivial importance, such as intensification of steroid therapy or hospital readmission, can lead to challenges in interpreting the meaning of a composite endpoint. A higher rate of treatment failure in the placebo group on the composite outcome could conceivably lead one to believe that the placebo group had higher rates of the more important endpoint of death. However, there were no differences between the groups for the most important endpoint of death. Over the six-month follow up, 9.9% (11 of 111) of patients receiving placebo and 8.1% (13 of 160) of patients receiving glucocorticoids died (p=0.61). By combining the important endpoint of death with a more frequently occurring and relatively less important endpoint such as increase in steroid intensity, the authors were able to state that there was a statistically significant difference in the “composite” endpoint that included the important endpoint of death, although it was the relatively unimportant endpoint of increasing steroid intensity that was responsible for pushing the composite over the threshold for statistical significance. The large gradient in importance between the components in this study should prompt clinicians to conclude that the composite should not be used as a basis for medical decision-making and instead focus on the individual component endpoints.

### Did the Component Endpoints Occur with Similar Frequency?

The larger the gradient in frequency between the most and least patient-important component endpoints, the more skeptical we should be about the usefulness of using the composite endpoint as a basis for medical decision-making. If the more important component endpoints occur with far less frequency than the less important ones, the composite endpoint becomes less informative.

In a prospective, randomized, multicenter trial reported in *The Lancet*, the authors evaluated the effectiveness of invasive vs. medical therapy in elderly patients (≥ 75 years) with chronic angina.^13^ The primary endpoint was quality of life after six months, as assessed by questionnaire and the presence of major adverse cardiac events (a composite endpoint of death, non-fatal myocardial infarction [MI], or hospital admission for acute coronary syndrome). The authors observed that angina severity decreased and quality of life measures increased in both groups, with improvements greater after revascularization. The authors also reported that major adverse cardiac events occurred more frequently in the medical group (49% vs. 19%, p<0.0001), and stated that patients aged 75 years or older with angina despite standard drug therapy benefit more from revascularization than from optimal medical therapy.

The difference in major adverse cardiac events of 30% between the medical and invasive group could conceivably lead a reader to believe that patients in the invasive group had lower rates of death, non-fatal MI, and hospital admission. However, the significant difference in the composite endpoint in this instance was solely due to an increased frequency of the least important outcome, hospital admissions, which accounted for 76% of events in the medical group, as compared to 36% of events in the invasive strategy group. This large gradient in frequency should prompt clinicians to focus on the individual component endpoints, and not the composite.

### Can One Be Confident that the Component Endpoints Share Similar Relative Risk Reductions?

The confidence clinicians can have regarding the similarity in relative risk reductions among the component endpoints can be evaluated with two questions:

1. Is the underlying biology of the component endpoints similar enough such that one would expect to see similar relative risk reductions?

The rationale for using a composite endpoint is in part dependent on the confidence clinicians can have that the more and less important component outcomes share similar relative risk reductions. The stronger the biologic rationale for why an intervention should have a particular effect on the component endpoints, the more confident clinicians can become with the notion that the composite endpoint accurately portrays the net effect of treatment.

2. Are the point estimates of the relative risk reductions similar and confidence intervals (CI) sufficiently narrow?

Although a strong biologic rationale supporting similar treatment effects across component endpoints is reassuring, it is the actual observation of similar treatment effects among the component endpoints that leads to increased confidence in using the composite endpoint as a basis for medical decision-making. The larger the gradient in results between the more and less important component endpoints, the larger should be our concern about using a composite endpoint. This is particularly true for composite endpoints with components that include both beneficial and harmful effects.

The Losartan Intervention For Endpoint reduction (LIFE) trial was a prospective double-blinded, randomized study that evaluated the effectiveness of a losartan-based vs. atenolol-based antihypertensive treatment regimen on the composite outcome of death, MI, or stroke, for patients aged 55–80 years of age with essential hypertension and left ventricular hypertrophy.^14^ The authors observed no significant difference in blood pressure reduction between the groups. They also reported a decreased risk in the primary composite endpoint in the losartan group (risk ratio [RR] [0.87], 95% CI [0.77 – 0.98]). Of the component endpoints, only the risk of stroke had a statistically significant reduction (RR [0.75], 95% CI [0.63 – 0.89]). The authors concluded that losartan prevents more cardiovascular morbidity and death than atenolol for a similar reduction in blood pressure, despite the lack of significant difference in death rates between the groups. The challenges for composite endpoint interpretation as well as the potential for widespread distribution of misleading study results is evidenced by the U.S. Food and Drug Administration restricting the regulatory labeling of the use of losartan for reduction of nonfatal stroke, as opposed to the original triple endpoint of death, MI, or stroke in the LIFE trial.^6^,^14^,^15^

## SUMMARY

Composite endpoints in clinical trials are composed of primary endpoints that contain two or more distinct component endpoints. The purported benefits include increased statistical efficiency, decrease in sample-size requirements, shorter trial duration, and decreased cost. However, the purported benefits must be diligently weighed against the inherent challenges in interpretation. Furthermore, the larger the gradient in importance, frequency, or results between the component endpoints, the less informative the composite endpoint becomes, thereby decreasing its utility for medical-decision making.

## Figures and Tables

**Figure:**
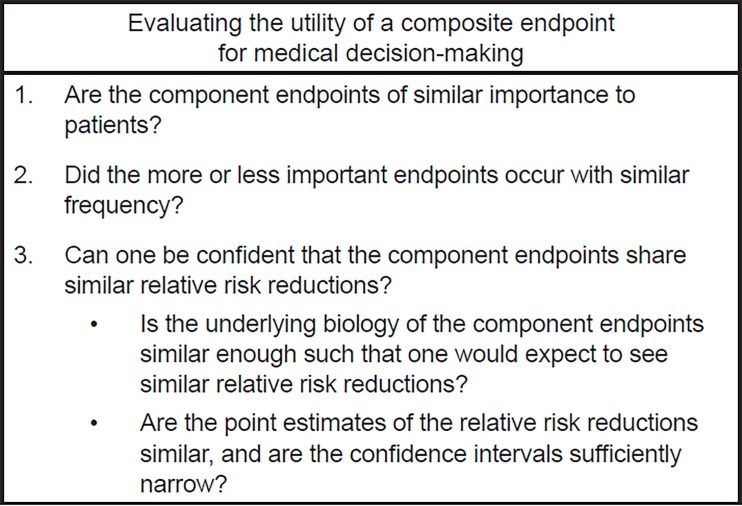
Questions to aid clinicians in evaluating the utility of a composite endpoint as a basis for medical decision-making (for therapies purported to decrease the risk of an undesirable outcome).
